# Myomerger induces fusion of non-fusogenic cells and is required for skeletal muscle development

**DOI:** 10.1038/ncomms15665

**Published:** 2017-06-01

**Authors:** Malgorzata E. Quinn, Qingnian Goh, Mitsutoshi Kurosaka, Dilani G. Gamage, Michael J. Petrany, Vikram Prasad, Douglas P. Millay

**Affiliations:** 1 Division of Molecular Cardiovascular Biology, Cincinnati Children's Hospital Medical Center, 240 Albert Sabin Way, Cincinnati, Ohio 45229, USA

## Abstract

Despite the importance of cell fusion for mammalian development and physiology, the factors critical for this process remain to be fully defined, which has severely limited our ability to reconstitute cell fusion. Myomaker (*Tmem8c*) is a muscle-specific protein required for myoblast fusion. Expression of myomaker in fibroblasts drives their fusion with myoblasts, but not with other myomaker-expressing fibroblasts, highlighting the requirement of additional myoblast-derived factors for fusion. Here we show that *Gm7325*, which we name myomerger, induces the fusion of myomaker-expressing fibroblasts. Thus, myomaker and myomerger together confer fusogenic activity to otherwise non-fusogenic cells. Myomerger is skeletal muscle-specific and genetic deletion in mice results in a paucity of muscle fibres demonstrating its requirement for normal muscle formation. Myomerger deficient myocytes differentiate and harbour organized sarcomeres but are fusion-incompetent. Our findings identify myomerger as a fundamental myoblast fusion protein and establish a system that begins to reconstitute mammalian cell fusion.

## Abstract (web summary)

Cellular fusion is fundamental for skeletal muscle development. Here the authors show that myomerger is expressed in myoblasts, is essential for myoblast fusion in mice, and in co-operation with myomaker confers fusogenic ability to non-fusogenic cells.

The fusion of plasma membranes is necessary for numerous biological processes from conception to the development of skeletal muscle, osteoclasts, trophoblasts and giant cells^1^. The molecular regulation of fusion is poorly understood and the reconstitution of fusogenicity has not been achieved with mammalian proteins. Specifically, the factors that directly participate in membrane coalescence have not been identified. The development of systems that reconstitute fusion in the absence of other processes allow identification of nodal fusion machinery and associated molecular mechanisms. For example, the *Caenorhabditis elegans* fusogen epithelial fusion failure (Eff-1) is sufficient to fuse typically non-fusing cells^2^,^3^ and mechanisms of intracellular membrane fusion were partially revealed through reconstitution of SNAREs on synthetic membranes^4^,^5^,^6^. Thus, discoveries of specific fusion proteins and development of reconstitution systems have been historically critical to decipher multiple types of membrane fusion, however these systems are lacking for mammalian cellular fusion.

Myoblast fusion is a highly regulated process essential for muscle formation during development and regeneration^7^. While numerous proteins have been shown to contribute to mammalian myoblast fusion^8^,^9^,^10^,^11^,^12^,^13^,^14^,^15^,^16^,^17^,^18^, myomaker is the only known muscle-specific protein absolutely required for this process^19^,^20^. Although its biochemical function is unknown, expression of myomaker in fibroblasts or mesenchymal stromal cells (MSCs) induces their fusion with muscle cells^21^,^22^. Myomaker-expressing fibroblasts do not fuse to each other indicating that these cells harbour a competency to fuse, but only in the presence of a fusogenic cell (such as muscle cell). Thus, additional myocyte factors that confer fusogenicity would be required for reconstitution of fusion in myomaker^+^ fibroblasts. This finding also suggests that unlike virus-cell fusion^23^ or *C. elegans* epithelial cell fusion^2^, which are both controlled by single factors, namely vesicular stomatitis virus (VSV) and Eff-1 respectively, mammalian muscle cell fusion is regulated by multiple proteins.

Using the idea that additional factors necessary for fusion would be expressed on myocytes but not fibroblasts, we discovered that *Gm7325* (myomerger) is sufficient to fuse myomaker-expressing fibroblasts. Cell mixing experiments reveal that while myomaker renders cells fusion competent, myomerger induces fusogenicity. We show that myomerger is exclusively expressed in skeletal muscle only during developmental and regenerative myogenesis. Disruption of myomerger in myoblast cell lines and in the mouse, through Cas9-mutagenesis, generates non-fusogenic myocytes. Our study shows that myomerger controls myoblast fusion and, together with myomaker, reconstitutes cell fusion.

## Results

### Identification and fusogenic activity of myomerger

**Figure 1:**
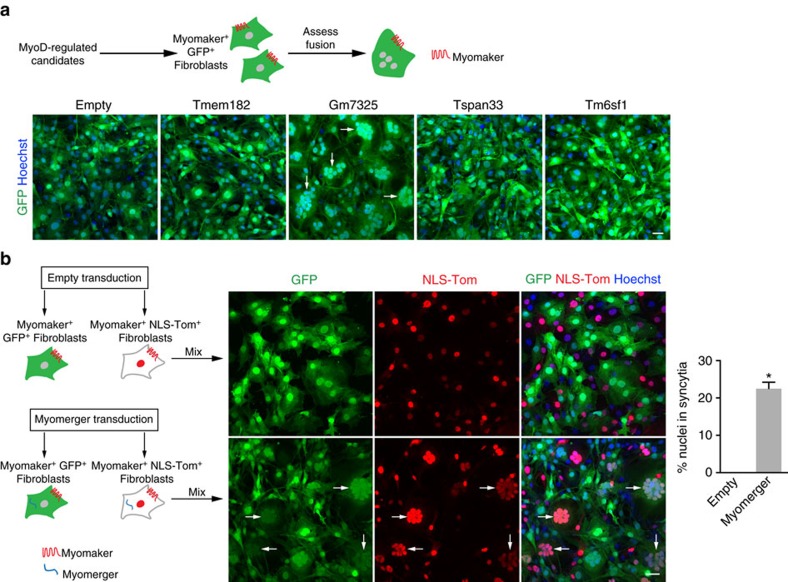
Induction of fibroblast fusion by myomerger. (**a**) Schematic showing a functional assay to screen for muscle genes that could activate fusion of GFP^+^ myomaker^+^ fibroblasts. Representative images of GFP^+^ cells and nuclei after expression of the indicated genes. Arrows depict cells with multiple nuclei. (**b**) Illustration of cell mixing approach to show fusion between the populations of fibroblasts. Co-localization of GFP and NLS-TdTomato (NLS-Tom) in the nucleus represents fusion. Representative images demonstrate fusion of myomaker^+^ myomerger^+^ fibroblasts but not empty-infected myomaker^+^ fibroblasts. Arrows indicate fusion between GFP^+^ and NLS-Tom^+^ fibroblasts. The percentage of nuclei in syncytia after expression of empty or myomerger (*n*=3). Data are presented as mean±s.e.m. *P<0.05 compared with empty using an unpaired *t*-test. Scale bars, 50 μm.

To uncover potential fusion factors, we compared genes induced by expression of MyoD to their level of expression in 10T ½ fibroblasts. Of the top 100 MyoD-regulated genes not expressed in fibroblasts (Supplementary Table 1) we eliminated genes not likely to be directly involved in fusion (transcription factors, sarcomeric and metabolic genes) and focused on genes with transmembrane domains. This analysis yielded the following five genes: *Tmem182*, *Gm7325*, *Cdh15*, *Tspan33* and *Tm6sf1*, however *Cdh15* was omitted from further analysis because it is not necessary for myoblast fusion or muscle formation^24^. We retrovirally expressed each gene in myomaker^+^ GFP^+^ fibroblasts and assayed for fusion. Appropriate expression in fibroblasts was verified through quantitative reverse transcription polymerase chain reaction (qRT-PCR) analysis (Supplementary Fig. 1). We observed mainly mono-nucleated GFP^+^ cells in all cultures except when *Gm7325* was expressed where widespread multi-nucleated cells were present (Fig. 1a). Based on the ability of *Gm7325* to induce fusion of myomaker^+^ fibroblasts and the observations described below we named the protein myomerger.

Multiple *Gm7325* transcripts are annotated in the University of California, Santa Cruz, mouse genome. The shorter transcript contains a single exon and yields a protein with 84 amino acids. In contrast, the longer transcript utilizes an upstream exon with an alternative start site and results in a protein of 108 amino acids (Supplementary Fig. 2a). The single coding exon of the short transcript is conserved in other mammalian genomes, including humans, while the upstream alternative exon leading to the longer transcript is not highly conserved (Supplementary Fig. 2b). For the initial screen we cloned the *Gm7325* locus into a retroviral vector, allowing normal splicing and expression of both short and long transcripts. Transduction of myomaker^+^ fibroblasts with either myomerger-short (S) or myomerger-long (L) induced formation of multi-nucleated cells, indicating both proteins are sufficient for fusion (Supplementary Fig. 2c). Additionally, myomerger and myomaker together induced fusion of 3T3 fibroblasts and MSCs (Supplementary Fig. 2d), suggesting these two genes could activate fusion in a multitude of cell types.

Given that multi-nucleated cells could arise from fusion or replication associated with incomplete cytokinesis, we designed a system to validate that multi-nucleated cells observed in fibroblasts expressing both myomerger and myomaker were generated through fusion. We engineered two fibroblast cell lines that both express myomaker, with one expressing GFP and the other expressing nuclear-localized TdTomato (NLS-Tom). Myomaker^+^ GFP^+^ and myomaker^+^ NLS-Tom^+^ fibroblasts were infected with a myomerger retrovirus or a control empty retrovirus, mixed, and fusion was assessed (Fig. 1b). We observed cells with multiple nuclei containing both GFP and NLS-Tom in fibroblasts expressing myomaker and myomerger indicating fusion (Fig. 1b). Quantification of fusion revealed approximately 20% of nuclei were contained in syncytia in cultures where fibroblasts were expressing both myomaker and myomerger (Fig. 1b). These results confirm that the observed syncytial cells are formed through fusion and that expression of myomaker and myomerger is sufficient to confer fusogenicity in non-fusogenic fibroblasts.

**Figure 2:**
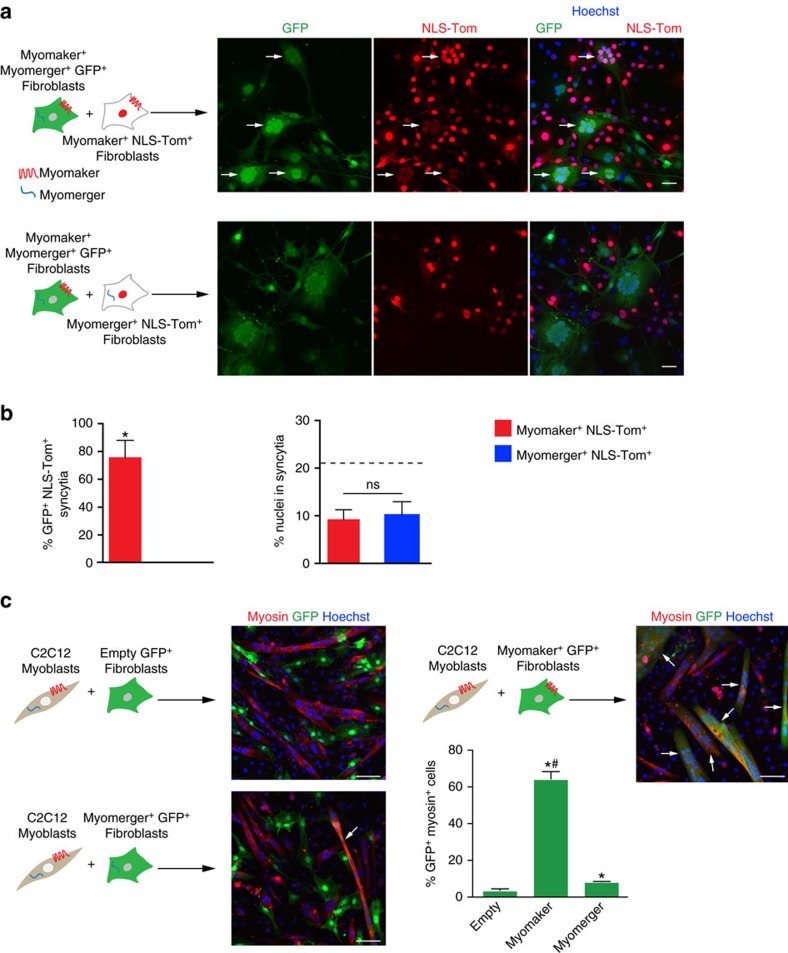
Efficient fusion requires myomaker expression in both fusing cells but myomerger in one fusing cell. (**a**) Diagram showing the cell mixing approach to assess fusion between the populations of fibroblasts. Co-localization of GFP and NLS-TdTomato (NLS-Tom) in the nucleus represents fusion (arrows). Representative images demonstrate fusion of myomaker^+^ myomerger^+^ GFP^+^ fibroblasts with myomaker^+^ NLS-Tom^+^ fibroblasts but not myomerger^+^ NLS-Tom^+^ fibroblasts. (**b**) Quantification of the percent of GFP^+^ NLS-Tom^+^ syncytia and the percent of nuclei in syncytia (*n*=3). Dotted line on right panel represents fusion achieved when both cells express both myomaker and myomerger (from Fig. 1b). (**c**) Heterologous fusion experiment between C2C12 myoblasts and GFP^+^ fibroblasts infected with either empty, myomaker or myomerger. Representative immunofluorescent images to visualize co-localization of myosin and GFP (arrows), indicating fusion. Quantification of the percentage of GFP^+^ myosin^+^ cells (*n*=3). Data are presented as mean±s.e.m. *P<0.05 compared with myomerger^+^ NLS-Tom^+^ fibroblasts in **b** or empty in **c**. #*P*<0.05 between myomaker and myomerger. An unpaired *t*-test was used to determine significance. Scale bars, 50 μm (**a**), 100 μm (**c**).

We also sought to determine the cell biology of fusion induced by myomaker and myomerger. We mixed myomaker^+^ myomerger^+^ GFP^+^ fibroblasts with NLS-Tom^+^ fibroblasts expressing myomaker or myomerger (Fig. 2a). Here we observed fusion of myomaker^+^ myomerger^+^ GFP^+^ fibroblasts with myomaker^+^ NLS-Tom^+^ but not myomerger^+^ NLS-Tom^+^ fibroblasts (Fig. 2a). We detected 10% of nuclei in syncytia (Fig. 2b), significantly lower than the fusion observed when both cells express myomaker and myomerger (Fig. 1b) suggesting an enhanced fusogenic efficiency when cells express both proteins. Nonetheless, these data indicate that myomerger is not sufficient for fusion in the absence of myomaker. This heterotypic nature of fibroblast fusion, where myomaker is required on both cells and myomerger is only required on one cell, are consistent with our previously reported heterologous fusion system between myoblasts and fibroblasts^19^. In that system, myomaker^+^ fibroblasts that do not express myomerger fused with muscle cells, which express both myomaker and myomerger. To confirm this concept, we utilized our heterologous fusion system where fibroblasts were infected with GFP and either empty, myomaker, or myomerger retrovirus, and then mixed with C2C12 myoblasts (Fig. 2c). In this assay, fusion is detected through co-localization of GFP (fibroblasts) with myosin^+^ myotubes. Compared with empty-infected GFP^+^ fibroblasts, we detected a significant increase in fusion between myosin^+^ cells with either myomaker^+^ GFP^+^ fibroblasts or myomerger^+^ GFP^+^ fibroblasts (Fig. 2c). However, quantification of myosin^+^ GFP^+^ cells revealed that myomerger did not drive the fusion of fibroblasts with muscle cells to the levels observed with myomaker (Fig. 2c). These data confirm myomaker is required in both fusing cells for *in vitro* fusion, while myomerger is essential in one fusing cell.

### Myomerger is muscle specific and associates with membranes

**Figure 3:**
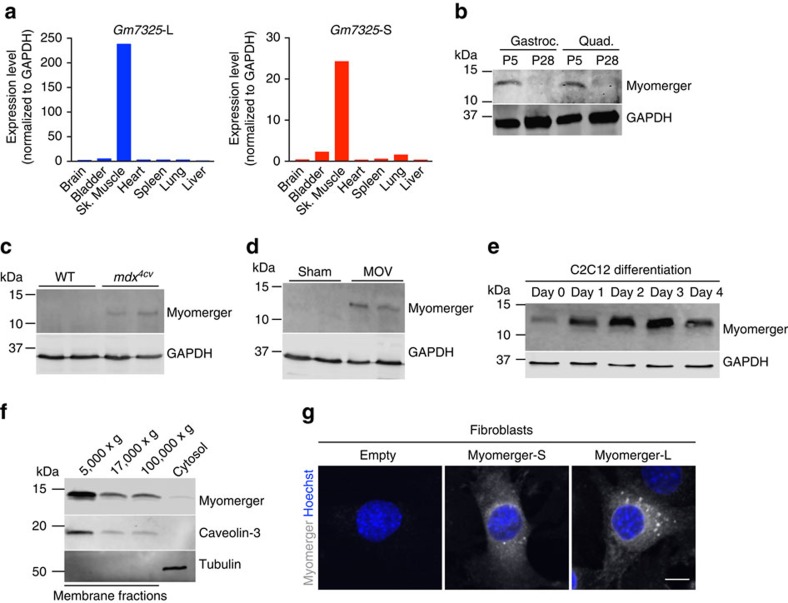
Muscle-specific expression and regulation of myomerger. (**a**) qRT-PCR for both *Gm7325* long (L) and short (S) transcripts from various postnatal (P) day 5 tissues. (**b**–**e**) Immunoblotting for myomerger comparing P5 muscle to P28 muscle (**b**), WT to *mdx*^*4cv*^ diaphragms (8 weeks of age) (**c**), sham plantaris to mechanically overloaded (MOV) plantaris (3 months of age) (**d**), and during C2C12 myoblast differentiation (**e**). GAPDH was used as a loading control. (**f**) Fractionation of C2C12 lysates on day 2 of differentiation followed by immunoblotting. (**g**) Representative immunostaining of fibroblasts infected with either empty, myomerger-short (S), or myomerger-long (L). Scale bar, 10 μm.

The only current information regarding *Gm7325* is its potential expression in embryonic stem (ES) cells^25^, therefore we interrogated its expression pattern more thoroughly. We performed qRT-PCR on multiple tissues from postnatal (P) day 5 mice with primers to distinguish the two potential mouse transcripts (Supplementary Fig. 3a). In neonatal tissues, we detected expression of both myomerger transcripts only in skeletal muscle (Fig. 3a). Despite the evidence of two myomerger transcripts, immunoblot analysis of skeletal muscle lysates from P5 mice using a commercially available antibody identified a single band at approximately 12 kDa. This band was absent in P28 lysates indicating that myomerger is downregulated after neonatal development (Fig. 3b). Skeletal muscle exhibits a robust ability to regenerate due to the presence of muscle stem cells, also known as satellite cells^26^,^27^. We analysed expression of myomerger in *mdx*^4cv^ mice, which is a mouse model of muscular dystrophy that leads to chronic cycles of degeneration and regeneration^28^. Myomerger expression was detected in diaphragm lysates from *mdx*^4cv^ mice, but not control diaphragms (Fig. 3c). Finally, we analysed expression of myomerger in a model of skeletal muscle overload-induced (MOV) hypertrophy and observed up-regulation (Fig. 3d). Collectively, these data demonstrate that myomerger is expressed only during development and is induced during adult myogenesis.

We next sought to determine if myomerger is regulated as myoblasts differentiate. In C2C12 cells, both *Gm7325* transcripts were significantly elevated during differentiation (Supplementary Fig. 3b). Similarly, myomerger protein levels were low in proliferating myoblasts (day 0), but increased upon differentiation with expression maintained during myoblast differentiation and fusion into myotubes (Fig. 3e). The short mouse myomerger protein, but not the long form, is highly conserved among vertebrate species (Supplementary Fig. 3c). After transduction of C2C12 cells with empty, myomerger-S, or myomerger-L we detected an increased upper band in cells expressing either myomerger-S or myomerger-L that co-migrated with the 12 kDa endogenous protein in the empty-infected C2C12 cells (Supplementary Fig. 3d). A lower band was identified exclusively in myomerger-S lysates suggesting that complex mRNA or post-translational processing results in the endogenous single 12 kDa band observed in WT C2C12 cells and muscle homogenates. Both myomerger proteins harbour a hydrophobic region close to the N terminus, where computational analysis of this region indicates a signal peptide or transmembrane domain (Supplementary Fig. 3e). Given that both variants were found to confer fusogenicity, the significance of the predicted domains is presently unclear. To understand subcellular localization, we fractionated C2C12 cells on day 2 of differentiation and identified myomerger in membrane fractions containing caveolin-3, a protein known to associate with both heavy and light vesicles (Fig. 3f). Immunostaining of fibroblasts expressing myomerger-S or myomerger-L shows that both proteins exhibit similar diffuse and vesicular localization (Fig. 3g). Thus, myomerger associates with membrane compartments consistent with its ability to induce fusion.

### Myomerger controls myoblast fusion

**Figure 4:**
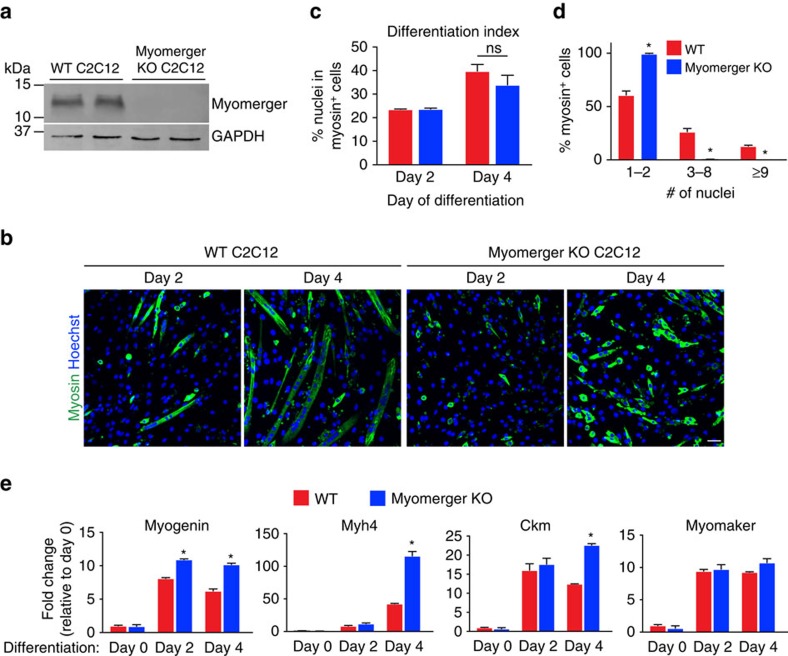
Requirement of myomerger for myoblast fusion *in vitro*. (**a**) Immunoblotting for myomerger in WT and myomerger KO C2C12 cells on day 2 of differentiation. GAPDH was used as a loading control. (**b**) Representative immunofluorescence images on day 2 and day 4 of differentiation for WT and myomerger KO C2C12 cells. Myomerger KO cells differentiate but fail to fuse. (**c**) Quantification of the differentiation index, the percentage of nuclei in myosin^+^ cells (*n*=4). NS, not significant. (**d**) The percentage of myosin^+^ cells that contain 1–2, 3–8, or ≥9 nuclei after 4 days of differentiation, as an indicator of fusogenicity (*n*=3). (**e**) qRT-PCR for the indicated myogenic transcripts (*n*=4). Data are presented as mean±s.e.m. *P<0.05 compared with WT using an unpaired *t*-test. Scale bar, 50 μm.

**Figure 5:**
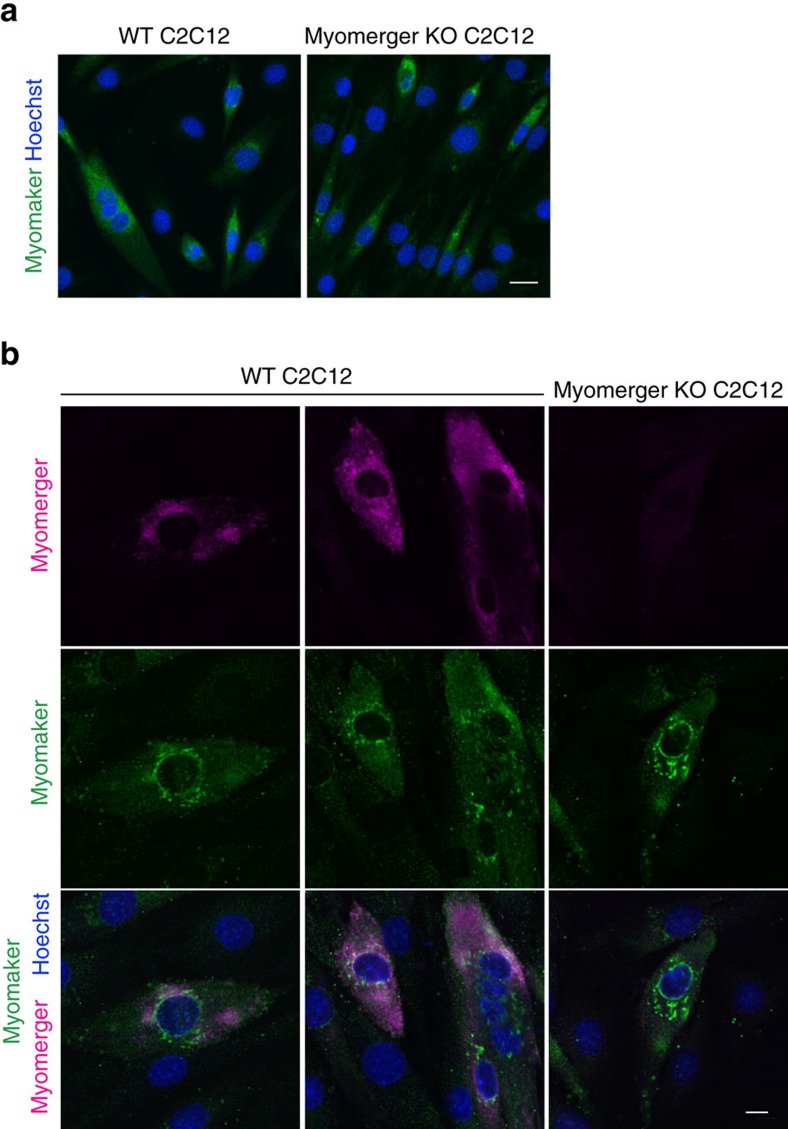
Analysis of myomaker and myomerger co-localization. (**a**) Representative immunofluorescence images from WT and myomerger KO C2C12 cells on day 2 of differentiation indicating that loss of myomerger does not alter myomaker expression or localization. (**b**) Immunofluorescence for myomerger and myomaker on the indicated cells on day 2 of differentiation. These two fusion proteins exhibit different localization patterns. Scale bars, 10 μm (**a**), 5 μm (**b**).

The ability of myomerger to induce fusion of myomaker-fibroblasts, and its muscle-specific expression in the mouse, suggests that myomerger may play a critical role during myogenesis. To begin to decipher the role of myomerger in myogenesis, we evaluated its function during myoblast differentiation. We utilized CRISPR/Cas9 genome editing to disrupt myomerger in C2C12 myoblasts. Two guide RNAs (gRNA) were designed to target the largest exon of *Gm7325*, which resulted in a 166 base pair deletion thereby disrupting both mouse transcripts (Supplementary Fig. 4a). C2C12 cells were transfected with a plasmid containing Cas9 with an IRES-GFP and myomerger gRNAs, or transfected with only Cas9-IRES-GFP as a control. Flow cytometry of GFP^+^ cells followed by genotyping through PCR analysis revealed disruption of the myomerger locus (Supplementary Fig. 4b). Myomerger was not detectable in myomerger KO C2C12 cells confirming efficient disruption of the locus (Fig. 4a). Control and myomerger KO C2C12 cells were then analysed for their ability to differentiate and form myotubes. WT myoblasts differentiated, as indicated by myosin^+^ cells, and fused to form multi-nucleated myotubes (Fig. 4b). In contrast, myomerger KO C2C12 cells exhibited the ability to differentiate but lacked fusogenic activity to form myotubes (Fig. 4b). Indeed, quantification of the differentiation index revealed no difference in the percentage of myosin^+^ cells between WT and myomerger KO cultures (Fig. 4c). Additionally, quantification of fusion demonstrated that myomerger KO myosin^+^ cells remain mono-nucleated while WT cells fuse (Fig. 4d). qRT-PCR analysis for the myogenic genes *Myogenin*, *Myh4*, *Ckm* and *Tmem8c* (myomaker) further indicated that myomerger KO myoblasts activate the differentiation program (Fig. 4e). Interestingly, myogenic transcripts were elevated in myomerger KO cells potentially suggesting a feedback mechanism by which non-fusogenic cells attempt to further differentiate (Fig. 4e). Infection of myomerger KO C2C12 cells with either myomerger-S or myomerger-L rescued the fusion defect demonstrating that the phenotype in these cells is specifically due to the loss of myomerger and not an off-target effect of Cas9 (Supplementary Fig. 4c). Western blot analysis from these lysates shows re-expression of myomerger in KO cells (Supplementary Fig. 4d). As a potential mechanism for the lack of fusion in myomerger KO myocytes, we examined expression and localization of myomaker. On day 2 of differentiation, myomerger KO cells exhibited normal expression and localization of myomaker (Fig. 5a). Moreover, we did not detect widespread co-localization between myomaker and myomerger suggesting that myomerger does not directly regulate myomaker distribution (Fig. 5b). These data reveal that myomerger is necessary for myoblast fusion *in vitro* through a mechanism that does not involve regulation of myomaker levels or localization.

### Myomerger is required for muscle formation *in vivo*

**Figure 6:**
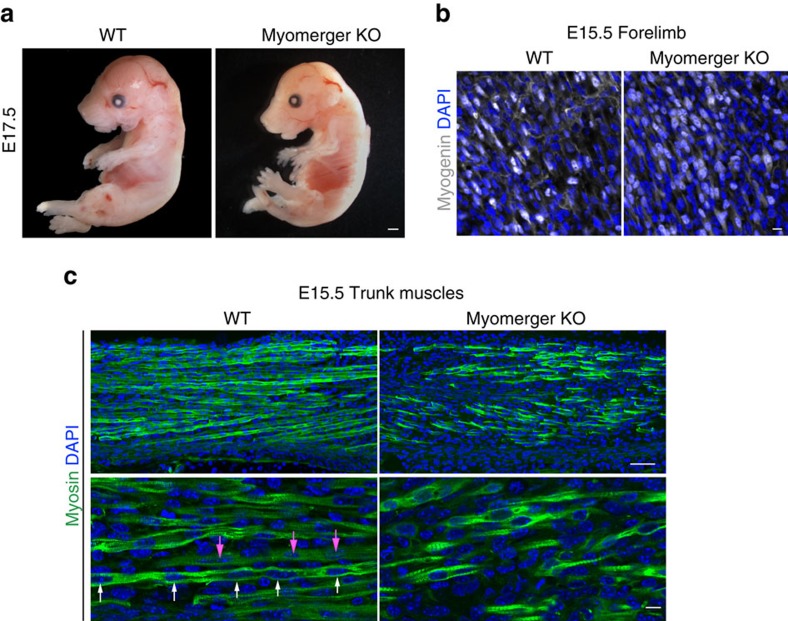
Myomerger is essential for myoblast fusion and muscle formation during embryonic development. (**a**) Representative whole-mount images of WT and myomerger KO E17.5 embryos showing improper skeletal muscle formation in KO embryos (*n*=4). (**b**) Immunofluorescence images for myogenin from WT and myomerger KO E15.5 forelimbs demonstrating that myomerger is not required for myogenic activation (*n*=3). (**c**) Myosin immunofluorescence on the indicated E15.5 trunk muscles (*n*=3). Multi-nucleated myofibres (arrows of same colour show nuclei within one myofibre) were observed in WT sections. Myomerger KO myocytes were myosin^+^ with sarcomeres but remained mono-nucleated. Scale bars, 1 mm (**a**), 50 μm (**c**), top panels, 10 μm (**b**,**c**), bottom panels.

To examine the function of myomerger *in vivo*, we disrupted exon 3 using the same CRISPR/Cas9 strategy described for C2C12 myoblasts. Injection of Cas9 and myomerger gRNAs into blastocysts resulted in lethality of 9 of the 10 F_0_ pups, suggesting that the high efficiency of Cas9 leads to homozygous deletion of myomerger. The one remaining pup was heterozygous for myomerger (Supplementary Fig. 5a) and sequencing of the mutant PCR product revealed the presence of the same mutation as was achieved in C2C12 cells. The heterozygous founder was mated to WT mice for multiple generations, which controlled for potential off-target effects given that we only selected pups with the *Gm7325* mutation. Heterozygous mice from these litters were then crossed to generate *Gm7325*^−/−^ mice. Although direct analysis for possible off-target effects of CRISPR/Cas9 deletion was not performed, an independently generated KO model using a unique gRNA strategy strongly supports the findings we describe below^29^. We failed to observe any *Gm7325*^−/−^ mice upon genotyping at P7 suggesting that myomerger is essential for life. Indeed, E17.5 *Gm7325*^−/−^ embryos exhibited minimal skeletal muscle upon gross examination (Fig. 6a). Specifically, bones of the limbs and rib cage were noticeable due to a scarcity of surrounding muscle. Myomerger KO mice also display a hunched appearance with elongated snouts, hallmark characteristics of embryos with improper muscle formation (Fig. 6a). Detection of myomerger by western blot of WT and *Gm7325*^−/−^ tongues showed elimination of myomerger protein in KO samples (Supplementary Fig. 5b). E15.5 forelimb sections show that myomerger KO myoblasts express myogenin indicating that specification and differentiation are activated despite loss of myomerger (Fig. 6b). Moreover, histological analysis of multiple muscle groups at E15.5 revealed the presence of myosin^+^ muscle cells and sarcomeric structures in myomerger KO mice (Fig. 6c; Supplementary Fig. 5c). While multi-nucleated myofibres were present in WT mice, these structures were not readily detected in myomerger KO mice indicating that genetic loss of myomerger renders myocytes non-fusogenic (Fig. 6c; Supplementary Fig. 5c). Analysis of forelimbs from E17.5 WT and myomerger KO embryos confirm that myomerger KO myoblasts are unable to properly fuse, although we did detect myocytes with two nuclei (Supplementary Fig. 5d–f). These results, together with our *in vitro* analysis, reveal that myomerger is required for muscle formation during mammalian development through regulation of myoblast fusion.

## Discussion

In summary, we report the discovery of an additional muscle-specific factor required for myoblast fusion and developmental myogenesis. While myomaker and myomerger are both necessary for muscle formation, E17.5 myomerger KO embryos grossly exhibit more myocytes compared with embryos lacking myomaker suggesting that these two key myoblast fusion proteins may have distinct functions. A recent publication suggests that myomaker and myomerger physically interact^30^, although we did not detect robust co-localization indicating that the potential interaction could be transient or occur at discrete cellular locations.

The precise biochemical function of these two fusion proteins is not known, however the fibroblast cell fusion system developed here, through expression of myomaker and myomerger, provides a unique platform to decipher these mechanisms. For example, our data from the cell mixing experiments reveal that myomaker is necessary in both fusing cells while myomerger is only required in one fusing cell. This indicates that, for reconstitution of cell fusion, both fusing cells must become fusion competent (amenable to fuse), while only one cell needs to become fusogenic to allow syncytial formation. With this concept in mind, our data suggest that myomaker allows the cell to become fusion competent, whereas myomerger confers fusogenicity. The cell mixing experiments also indicate that myomerger requires myomaker activity for fusogenic function because myomerger-expressing fibroblasts do not fuse to fibroblasts expressing both myomaker and myomerger. These data potentially suggest that myomerger acts downstream of myomaker, where myomaker acts as an initiator of fusion and myomerger executes the final steps to drive syncytial formation.

The distinct consequences of myomaker and myomerger expression in fibroblasts are consistent with the idea that the two proteins regulate different aspects of fusion. Membrane fusion is a complex process that includes membrane apposition and tethering, mixing of the outer membranes (hemifusion), pore formation, and pore expansion. Classical viral fusogens, as well as Eff-1, are large proteins with long ectodomains that are able to accomplish all of the steps necessary for fusion. In contrast, the discovery of myomerger as an additional myoblast fusion factor indicates that in higher organisms these multiple functions of viral proteins have been delegated to different myocyte proteins. This evolutionary strategy, at least in the case of muscle fusion, could provide more regulatory control to ascertain that cells are compatible for fusion.

How a relatively small protein, such as myomerger, induces membrane fusion is not understood although there is some precedence for small proteins activating fusion. Indeed, fusion-associated small transmembrane (FAST) proteins are a class of nonenveloped viral proteins that induce syncytium formation^31^. Given that myomerger associates with membranes it is tempting to speculate that it functions to alter membrane dynamics that overcomes the thermodynamic barriers for fusion. Another intriguing possibility, proposed by the data from the Sampath group^29^, is that myomerger activates fusion through cytoskeletal alterations. This potential function for myomerger would be consistent with induction of fusogencity as cytoskeletal alterations provide the necessary tension to induce membrane fusion in various systems^32^,^33^. Our identification of a second fusogenic factor reveals the complexity of plasma membrane fusion in mammalian cells, and highlights the potential for additional discoveries through utilization of the fusion reconstitution system achieved through co-expression of myomaker and myomerger.

## Methods

### Cell culture

C2C12 cells, 10T 1/2 fibroblasts, and NIH/3T3 fibroblasts were purchased from American Type Culture Collection and propagated in DMEM (Gibco) containing 10% heat-inactivated bovine growth serum (BGS) and supplemented with antibiotics. C2C12 cells were differentiated by switching to media containing 2% heat-inactivated horse serum (HS) and antibiotics. MSCs were a gift from Jose Cancelas^34^.

### Bioinformatic analysis

Microarray data from the GEO DataSet GSE34907 (ref. 35) was interrogated using GEO2R analysis to identify 1,826 genes displaying an increase greater than 1 log fold-change in MyoD-expressing fibroblasts. In parallel, a transcriptional profile of 10T 1/2 fibroblasts transduced with empty virus was generated using RNA-seq analysis (paired-end library layout using Illumina sequencing platform) and a list of all genes with RPKM values below 1.5 compiled using Strand NGS software (Ver. 2.6; Build: Mouse mm10 (UCSC) using Ensembl transcript annotations). These two gene lists were then compared with generate a final tally comprised of 531 genes that were both upregulated in MyoD-expressing fibroblasts and had low or no detectable expression in 10T 1/2 fibroblasts. Finally, the top 100 genes were interrogated for genes that contain transmembrane domains and not previously studied for their role during myoblast fusion.

### Animals

We used a dual sgRNA targeting strategy to create *Gm7325*^−/−^ mice. We selected the sgRNAs according to the on- and off-target scores from the web tool CRISPOR^36^. The selected gRNAs were 5′-GCAGCGATCGAAGCACCATC-3′ and 5′-GAGGCCTCTCCAGAATCCGG-3′ that target exon 3 of *Gm7325*. The sgRNAs were *in vitro* synthesized using the MEGAshortscript T7 kit (ThermoFisher) and purified by the MEGAclear Kit (ThermoFisher). sgRNAs (50 ng μl^−1^ of each) were mixed with 100 ng μl^−1^ Cas9 protein (ThermoFisher) and incubated at 37 °C for 15 min to form a ribonucleoprotein complex. We then injected the mix into the cytoplasm of one-cell-stage embryos of the C57BL/6 genetic background using a piezo-driven microinjection technique^37^. Injected embryos were immediately transferred into the oviductal ampulla of pseudopregnant CD-1 females. Live born pups were genotyped by PCR with primers spanning the mutated region (Supplementary Table 2). The edited allele was further confirmed by Sanger sequencing. One heterozygous founder was obtained and mated with WT C57Bl6 mice to eventually generate KO mice. The gender of analysed embryos was not determined. *Mdx*^*4cv*^ mice were purchased from Jackson Laboratory (#002378) and male mice were used. Muscle overload of the plantaris muscle was achieved through bilateral synergistic ablation of soleus and gastrocnemius muscles. Specifically, the soleus and gastrocnemius muscles were exposed by making an incision on the posterior-lateral aspect of the lower limb. The distal and proximal tendons of the soleus, lateral and medial gastrocnemius were subsequently cut and carefully excised. All animal procedures were approved by Cincinnati Children's Hospital Medical Center's Institutional Animal Care and Use Committee.

### CRISPR-mediated genome editing in C2C12 cells

Freshly plated low passage C2C12 cells were transfected with 4 μg of a modified pX458 plasmid (Addgene #48138, gift from Yueh-Chiang Hu), which contained a high fidelity Cas9, an optimized sgRNA scaffold, and an IRES-GFP cassette. The same gRNAs used to generate KO animals were used for C2C12 cells. 16 μl of Lipofectamine 2,000 was used for this transfection. 5 × 10^5^ C2C12 cells were transfected in a 60 mm culture dish. Forty-eight hours after transfection GFP^+^ cells were sorted into 96 well plates using FACS. These cells were maintained in DMEM containing 20% FBS with antibiotics at subconfluent densities. The cell lines were genotyped by amplifying a 420 bp region surrounding the site of Cas9 activity using the primers used to genotype *Gm7325*^−/−^ animals.

### Cloning and viral infection

We initially cloned a region of the *Gm7325* locus, containing all genomic information for expression of myomerger-short and myomerger-long, from C57Bl6 mouse genomic DNA. We cloned myomerger-short and long coding sequences from cDNA of differentiating C2C12 cells. Cloning primers are listed in Supplementary Table 2. Myomerger cDNA and genomic DNA were cloned into the retroviral vector pBabe-X^19^ using EcoRI. Myomaker and GFP retroviral plasmids have been described previously^19^. NLS-TdTomato was subcloned from pQC-NLS-TdTomato (Addgene #37347) into the retroviral vector pMX (Cell Biolabs). Plasmids containing cDNA for Tmem182, Tspan33, and Tm6sf1 from the Mammalian Gene Collection were purchased from Open Biosystems and subcloned into pBabe-X. Ten micrograms of retroviral plasmid DNA were transfected with FuGENE 6 (Roche) into Platinum E cells (Cell Biolabs), which were plated 24 h before transfection on a 10 cm culture dish at a density of 3–4 × 10^6^ cells per dish. Forty-eight hours after transfection, viral media were collected, filtered through a 0.45 μm cellulose syringe filter and mixed with polybrene (Sigma) at a final concentration of 6 μg ml^−1^. Target cells were plated on 10 cm culture dishes at a density of 4 × 10^5^ cells per dish 16–18 h before infection. Eighteen hours after infection, virus was removed, cells were washed with PBS and split into new 10 cm dishes.

### Cell fusion assays

Cells were split 18 h after retroviral infection and split again 24–48 h later. At the second split, cells were seeded for the fusion assay on 35-mm dishes (3–4 × 10^5^ cells per dish) or on 8-well Ibidi slides (2 × 10^4^ cells per well). Fusion was assessed 24–48 h after seeding. For heterologous fusion, cultures of fibroblasts and myoblasts mixed at a ratio of 1:1 (1.5 × 10^5^ cells for each) were induced to differentiate 24 h after seeding and fusion was assessed on day 4 of differentiation.

### RNA extraction and quantitative RT-PCR

Total RNA was extracted from either mouse tissue or cultured cells with TRIZOL (Life Technologies) according to manufacturer's protocol. cDNA was synthesized using High-Capacity cDNA Reverse Transcription Kit (Applied Biosystems) with random primers. Gene expression was assessed using standard quantitative PCR approach with Power Sybr Green PCR mastermix (Applied Biosystems). Analysis was performed on StepOnePlus Real-Time PCR system (Applied Biosystems) with gene-specific primers (Supplementary Table 2).

### Western blot analysis

Cultured cells were washed two times with ice cold PBS, scraped into a conical tube, pelleted, resuspended in lysis buffer (50 mM Tris-HCl, pH 6.8, 1 mM EDTA, 2% SDS) and sonicated for a total of 15 s (three 5 s pulses). Skeletal muscle tissues from mice were homogenized with a bead homogenizer (TissueLyser II; Qiagen) in lysis buffer (10 mM Tris (pH 7.4), 1 mM EDTA, 1 mM dithiothreitol, 0.5% Triton X-100, 2.1 mg ml^−1^ NaF) containing protease and phosphatase inhibitor cocktails (5 μl ml^−1^; Sigma-Aldrich). Both cells and tissue lysates were centrifuged to pellet insoluble material and protein concentration was determined using Bradford protein assay. Equal amounts of protein (5 μg for cells and 20 μg for tissues) were prepared with loading buffer (1 × Laemmli (Bio-Rad) with reducing agent (5% β-mercaptoethanol for cells and 100 mM DTT for tissues). Samples were heated at 37 °C for 30 min and separated on a 20% SDS-PAGE. The gels were subsequently transferred to a PVDF membrane (Millipore), blocked in 5% milk in Tris-buffered saline/0.1% Tween-20 (TBS-T) and incubated with sheep ESGP antibody (1 mg μl^−1^; R&D Systems) overnight at 4 °C. Membranes were then washed with TBS-T and incubated with Alexa-Fluor 647 donkey anti-sheep secondary antibody (1:5,000; Invitrogen). Bands were visualized using the Odyssey infrared detection system (LI-COR Biosciences). GAPDH (1:5,000; Millipore) was used as a loading control. Uncropped scans are provided in Supplementary Figs 6 and 7.

### Subcellular fractionation

C2C12 cells were harvested on day 2 of differentiation in ice cold hypotonic buffer (10 mM Tris–HCl pH 8, 2 mM EDTA) and lysed using a dounce homogenizer. Lysates were then centrifuged at 800 *g* for 5 min at 4 °C to separate nuclei and cell debris. That supernatant was then centrifuged at 5,000 *g* for 10 min to pellet mitochondria and ER. ER and heavy vesicles were further pelleted through centrifugation at 17,000 *g* for 10 min. Finally, plasma membrane, light vesicles, and organelles were pelleted at 100,000 *g* for 20 min and the supernatant from this spin was collected as the cytosolic fraction. All pellets were resuspended in lysis buffer (50 mM Tris-HCl, pH 6.8, 1 mM EDTA, 2% SDS) at volumes equal to the supernatant. 8 μl of each fraction was separated by SDS-PAGE and analysed for presence of myomerger, caveolin-3, and tubulin. Caveolin-3 antibody (BD Transduction Laboratories #610421) was used at 1:6,700 and tubulin (Santa Cruz #SC-8035) at 1:50.

### Immunocytochemistry

Cultured cells were rinsed with PBS and fixed in 4% paraformaldehyde (PFA)/PBS for 15 min at room temperature. Cells were subsequently permeabilized and blocked in 0.01% Triton X-100/5% donkey serum/PBS for one hour at room temperature. Primary antibody diluted in permeabilization/blocking buffer was incubated overnight. Cells were then washed with PBS and incubated with secondary Alexa-Fluor antibodies (1:250) for 1 h. A myomaker custom antibody was generated through YenZym Antibodies LLC. Rabbits were immunized with amino acids #137-152 of mouse myomaker (MKEKKGLYPDKSIYTQ) after conjugation to KLH. We used antigen-specific affinity purified products at a concentration of 4.3 μg ml^−1^ for immunostaining. Esgp (myomerger) antibody was used at a concentration of 1 μg ml^−1^. Anti-mouse myosin (my32, MA5-11748, ThermoFisher Scientific) antibody was used at 1:100. Hoechst 33342 solution (ThermoFisher Scientific) was used to stain nuclei. Cells were imaged using Nikon A1R+ confocal on a FN1 microscope (35 mm dishes) or Nikon A1R confocal on Eclipse T1 inverted microscope (Ibidi slides).

### Histology and immunohistochemistry

For cryosections, embryos were dissected, fixed in 4% PFA/PBS overnight at 4 °C, washed in PBS, incubated in 30% sucrose/PBS overnight and then in 1:1 mix of optimal cutting temperature (O.C.T.) formulation and 30% sucrose before embedding in O.C.T. Sections were cut at 10 μm and then permeabilized with 0.2% Triton X-100/PBS, blocked with 1% BSA/1% heat inactivated goat serum/0.025% Tween20/PBS and incubated with primary antibody overnight. Anti-mouse myosin (my32, MA5-11748, ThermoFisher Scientific) antibody was used at 1:100, whereas myogenin (F5D, Developmental Hybridomas) was used at a concentration of 2.56 μg ml^−1^. Secondary goat anti-mouse IgG1-488 Alexa-Fluor antibody (Invitrogen) was incubated at a dilution of 1:250 for 1 h. Slides were mounted with VectaShield containing DAPI (Vector Laboratories) and visualized using Nikon A1R confocal on Eclipse T1 inverted microscope. Images were analysed with Fiji.

### Statistical analysis

For quantitation of cell fusion in Figs 1b and 2b, cells with 3 or more nuclei were considered syncytial cells. The number of nuclei in syncytial cells and total number of nuclei were manually counted. To quantify fusion between myomaker^+^ myomerger^+^ GFP^+^ fibroblasts with either myomaker^+^ NLS-Tom^+^ or myomerger^+^ NLS-Tom^+^ fibroblasts (Fig. 2a), we calculated the percentage of GFP^+^ NLS-Tom^+^ syncytial cells. In Fig. 2c, the number of myosin^+^ myotubes (myosin structures with 3 or more nuclei) and GFP^+^ myosin^+^ myotubes were manually counted. The differentiation index (Fig. 4c) was calculated as the percentage of nuclei in myosin^+^ cells, and the fusion index (Fig. 4d) as the percentage of myosin^+^ cells with the indicated number of nuclei. For Supplementary Fig. 4c fusion was expressed as the percentage of myosin^+^ cells with ≥3 nuclei. Quantitative data sets are presented as means±s.e.m. For each quantitiation, at least 3 independent experiments were performed in duplicate and 4–6 fields were randomly chosen for imaging. Histological analysis of embryos was performed on 3–4 embryos per genotype per time point. Multiple histological levels within each muscle were examined. The data were analysed using an unpaired Student's *t*-test (two-tailed) with GraphPad Prism 6 software. A value of *P*<0.05 was considered statistically significant.

### Data availability

All relevant data are available from the authors on request. Accession code for the RNA sequencing analysis on 10T1/2 fibroblasts is GSE97361.

## Additional information

**How to cite this article:** Quinn, M. E. *et al*. Myomerger induces fusion of non-fusogenic cells and is required for skeletal muscle development. *Nat. Commun.* **8,** 15665 doi: 10.1038/ncomms15665 (2017).

**Publisher's note:** Springer Nature remains neutral with regard to jurisdictional claims in published maps and institutional affiliations.

## Supplementary Material

Supplementary Information Supplementary figures and supplementary tables.
