# Supplementary material for: Myomerger induces fusion of non-fusogenic cells and is required for skeletal muscle development
Source: Nat Commun. 2017 Jun 1;8:15665. doi: 10.1038/ncomms15665 (PMC5461499; doi:10.1038/ncomms15665)
Supplement: Supplementary Information — Supplementary figures and supplementary tables. [file ncomms15665-s1.pdf]

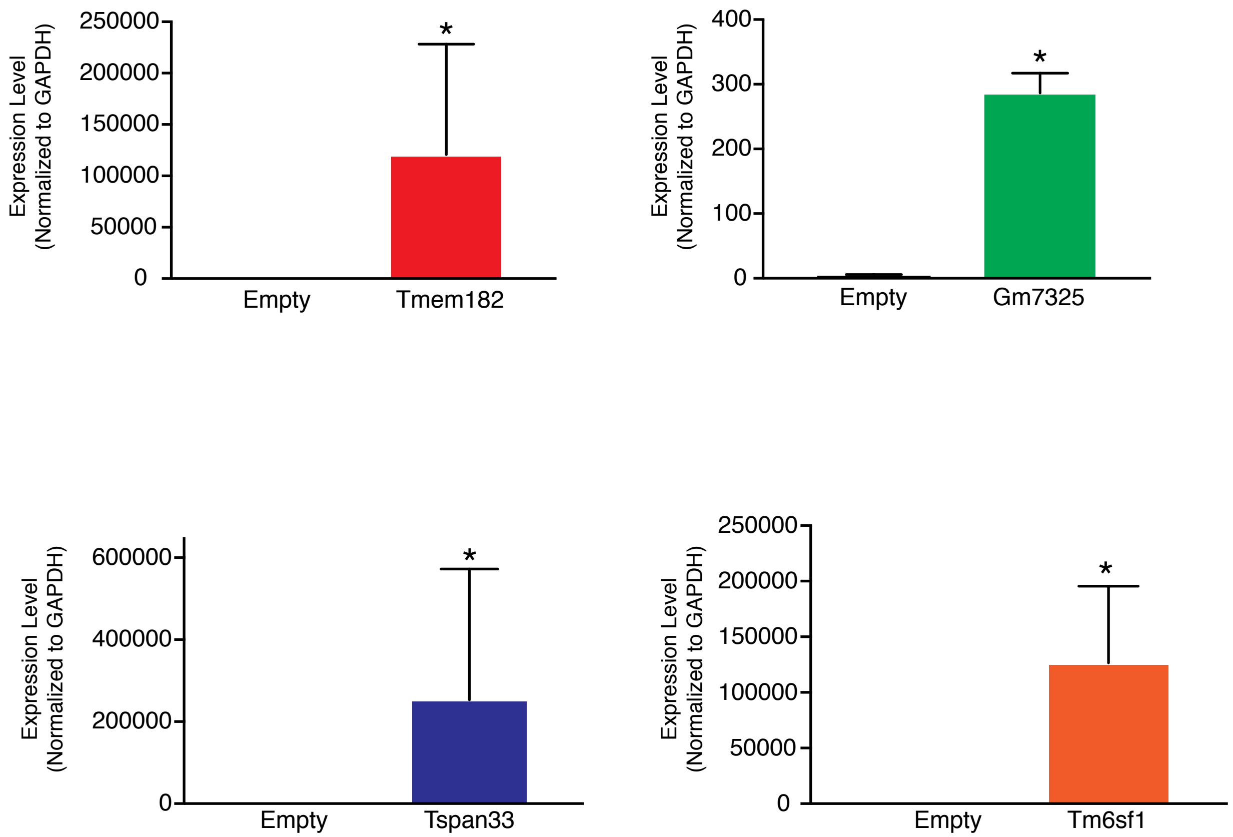

Supplementary Figure 1. Expression of MyoD-regulated genes in myomaker$^+$ fibroblasts. qRT-PCR analysis for the indicated genes 72 hours after expression in fibroblasts. For *Gm7325*, we used primers specific for the long transcript. Data are presented as mean ± SEM. *P<0.05 compared to Empty using an upaired t-test.

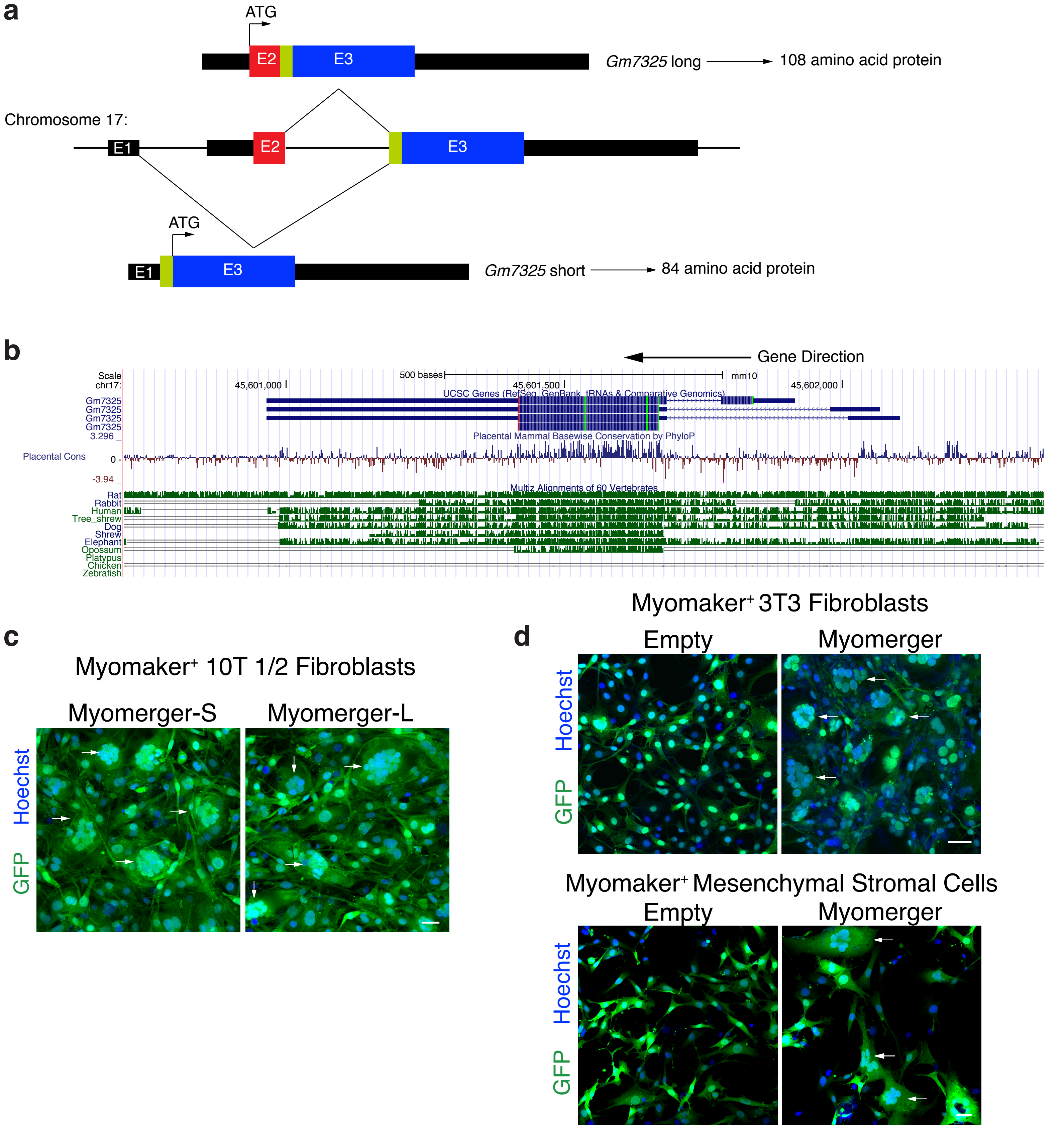

Supplementary Figure 2. Genomic organization of murine *Gm7325* and the ability of both variants to induce fusion. (**a**) Diagram showing the *Gm7325* locus on chromosome 17. The short transcript is generated by splicing of exon 1 (non-coding) with exon 3, leading to an 84 amino acid protein. The long transcript is produced by splicing of exon 2 with exon 3 and results in a 108 amino acid protein. (**b**) UCSC genome browser track showing multiple transcripts and conservation across vertebrate species. The short transcript is highly conserved in multiple species, including human, but not present in zebrafish. The upstream exon that produces the longer transcript is not highly conserved. Note that this annotation displays the gene on the reverse strand. (**c**) The short (S) or long (L) myomerger transcripts were expressed in myomaker+ 10T ½ fibroblasts and both induced fusion (*n*=3). (**d**) Myomerger also induced fusion of myomaker+ NIH/3T3 fibroblasts (*n*=3) and myomaker+ mesenchymal stromal cells (*n*=3). Arrows indicate fusion. Scale bars, 50 µm.

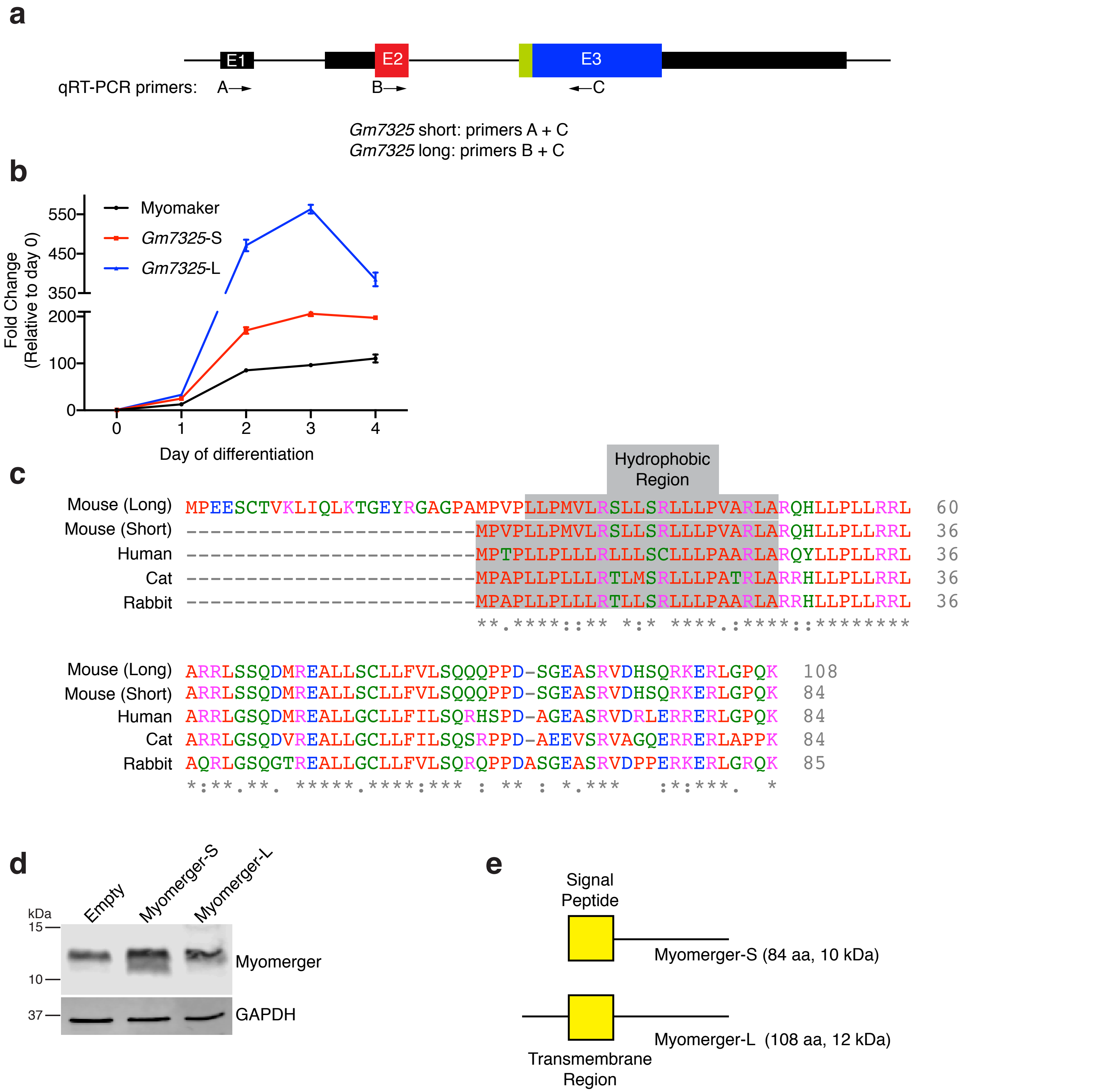

Supplementary Figure 3. Design of qRT-PCR primers and comparison of myomerger protein variants. (**a**) Schematic showing the location of primers to distinguish short and long transcripts. (**b**) qRT-PCR for *Gm7325* transcript variants and myomaker in C2C12 cells on the indicated days of differentiation (*n*=3 for each time point). (**c**) Sequence alignment of both mouse myomerger protein products with multiple mammalian orthologs using Clustal Omega. A potential hydrophobic region is highlighted in gray. (**d**) Immunoblotting from C2C12 cells infected with either empty, myomerger-short (S), myomerger-long (L) on day 2 of differentiation. Myomerger migrates as a single band around 12 kDa when endogenously produced (empty). Over-expression of myomerger-S leads to an increase in the endogenous band and a lower band is also detected suggesting that myomerger transcripts may be subjected to intricate mRNA processing or post-translational modifications. (**e**) Graphic showing the regions of myomerger-S and myomerger-L as predicted by SignalP and Phobius.

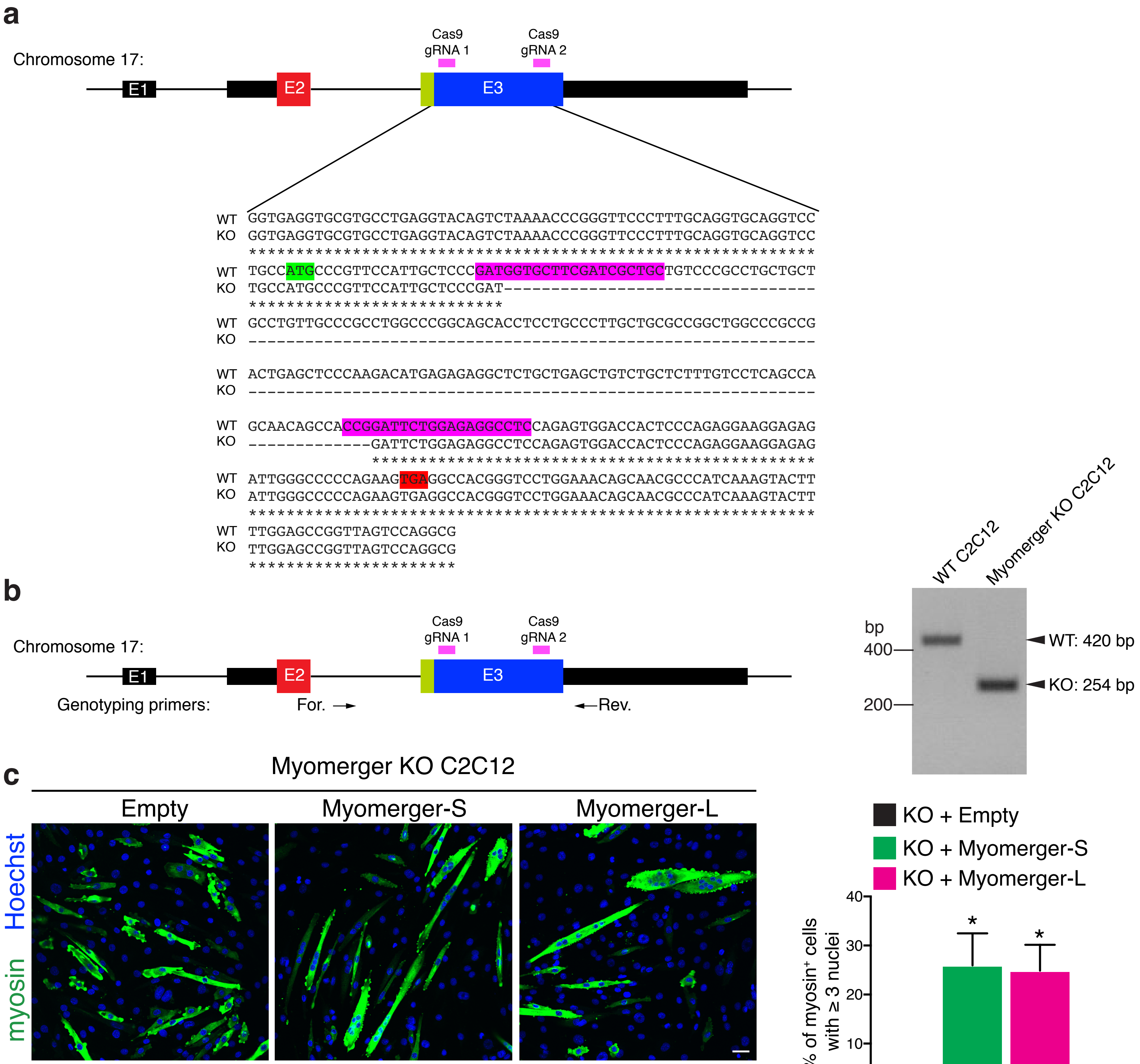

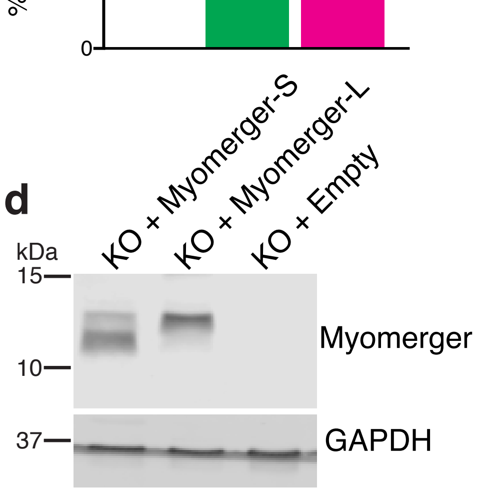

Supplementary Figure 4. CRISPR/Cas9 disruption of the *Gm7325* locus. (**a**) Schematic showing the Gm7325 locus and targeting of sgRNAs. (**b**) Genotyping strategy for myomerger KO C2C12 cells. WT and KO PCR products were sequenced and the result is shown below the diagram in **a**. The use of two sgRNAs results in reproducible cut sites leading to a 166 base pair deletion in both C2C12 cells and mice. The translational start site (ATG, green) for myomerger-S and stop site (TGA, red) for both myomerger-S and myomerger-L are noted. (**c**) Myomerger KO C2C12 cells were infected with either empty, myomerger-S, or myomerger-L and induced to differentiate. Both myomerger-S and myomerger-L rescued the lack of fusion in myomerger KO cells. Quantification of the fusion index, calculated as the percentage of myosin^{+} cells with ≥ 3 nuclei. Data are presented as mean ± SEM. **P*<0.05 compared to Empty using an upaired t-test. (**d**) Immunoblotting for myomerger shows appropriate expression after transduction of myomerger KO cells. Scale bars, 50 µm.

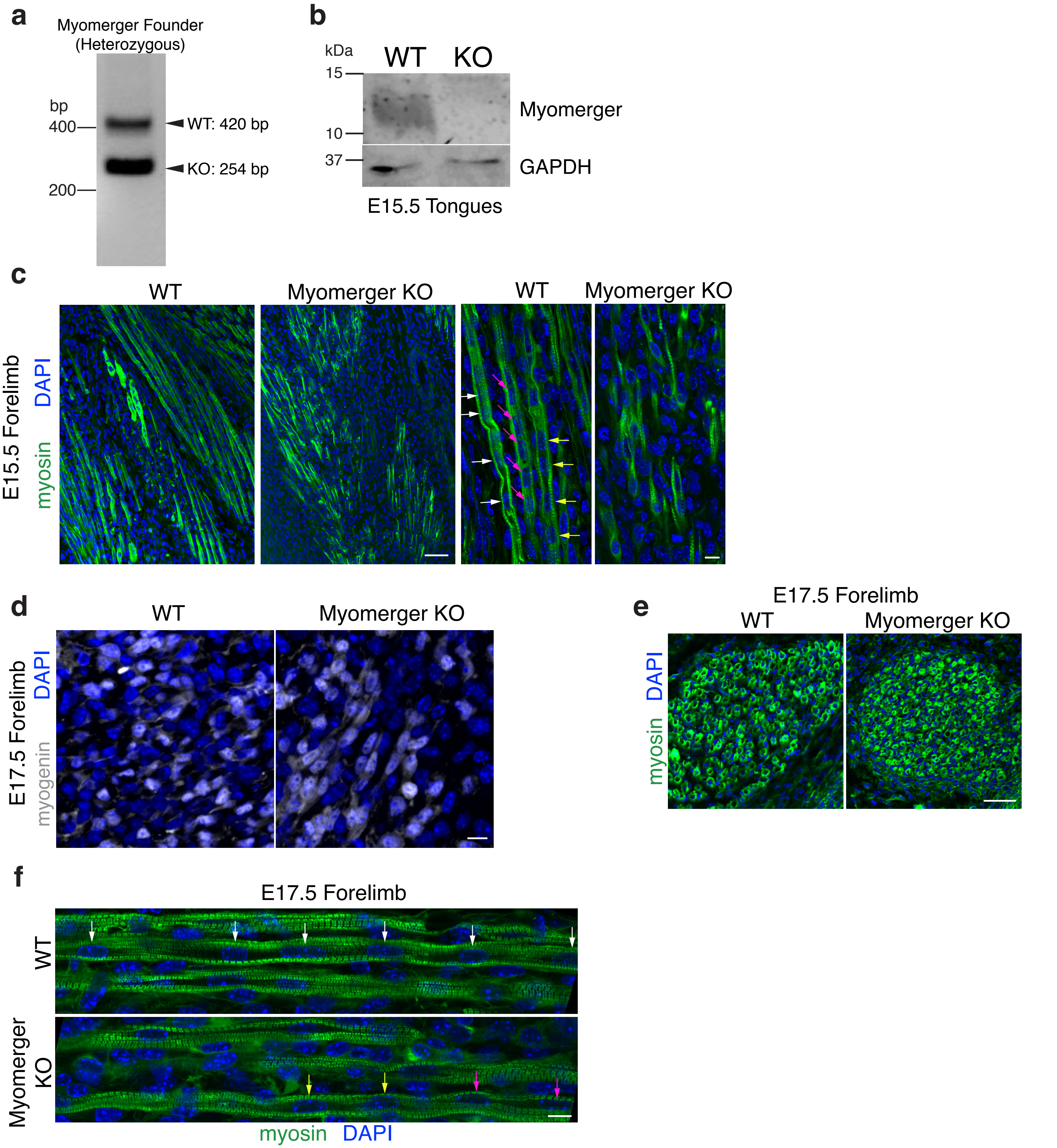

Supplementary Figure 5. Examination of myomerger KO muscle. (**a**) Genotyping of the one founder harboring the *Gm7325* mutation generated through Cas9-mutagensis. (**b**) Immunoblotting on tongue lysates from WT and myomerger KO mice showing lack of myomerger in KO samples. GAPDH was used as a loading control. (**c**) E15.5 forelimbs (*n*=3) immunostained with a myosin antibody demonstrates that myomerger KO myoblasts differentiate but are unable to fuse. Arrows of same color show nuclei within same fiber (**d-f**) E17.5 forelimbs from WT and myomerger KO mice were evaluated for myogenin and myosin expression, and multi-nucleation. Arrows of same color in **f** show nuclei within one myofiber. We observed myocytes in myomerger KO samples that contained two nuclei (arrows). The nuclei labeled by the yellow and pink arrows are within different myofibers. Scale bars, 50 µm **c,** left panels**, e**, 10 µm **c**, right panels, **d**, **f**.

Fig. 2b

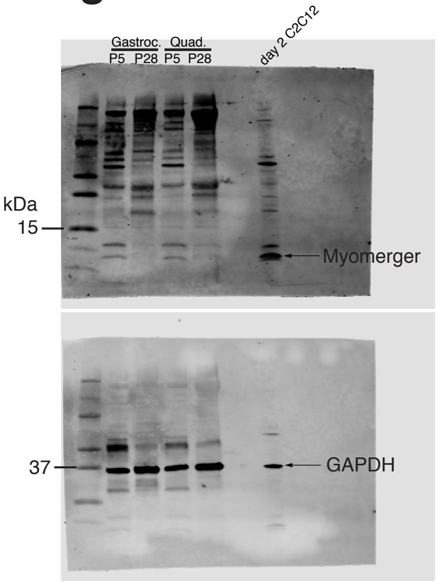

Fig. 2c and 2d

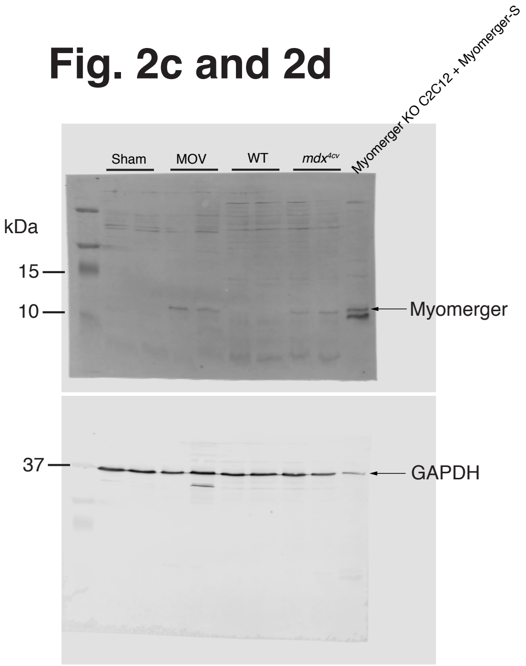

Fig. 2e

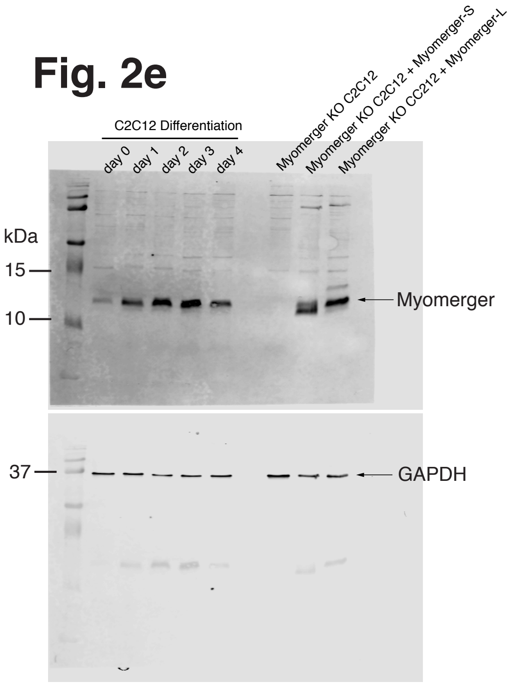

Fig. 2f

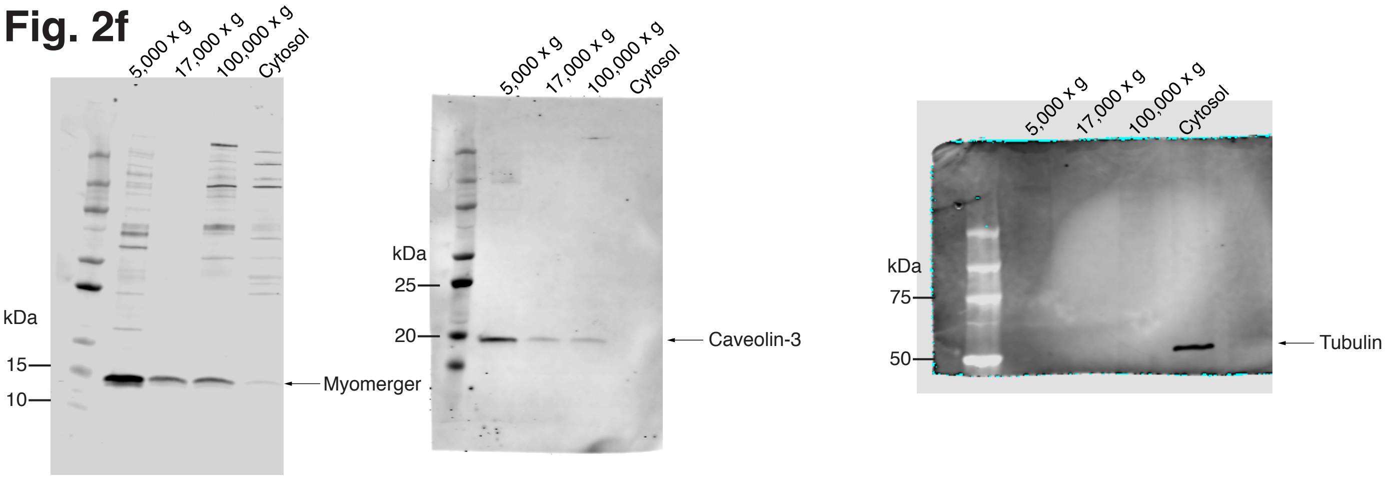

Fig. 3a

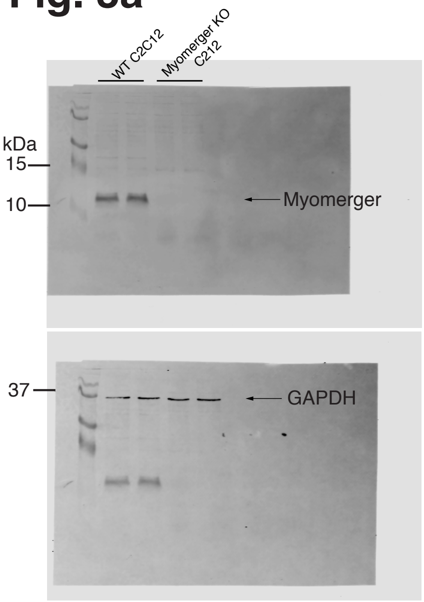

Supplementary Fig. 3d

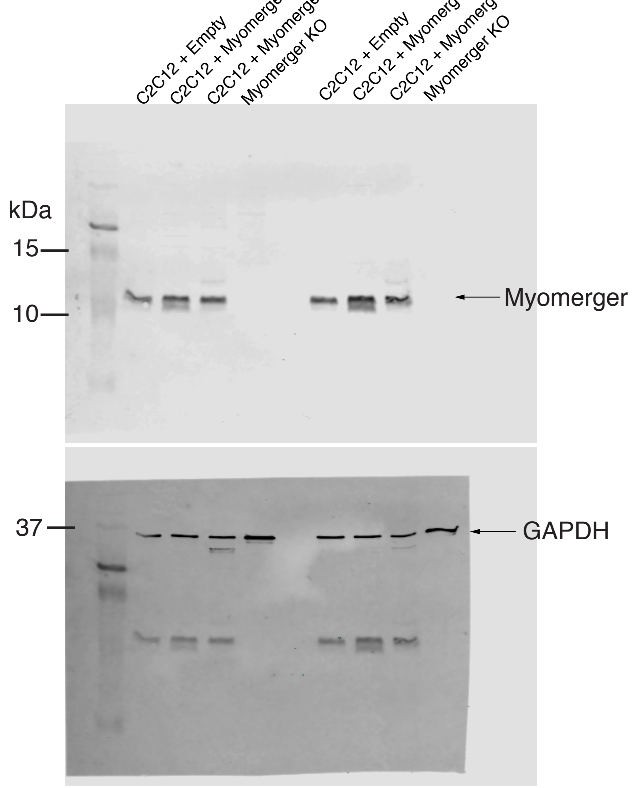

Supplementary Fig. 4d

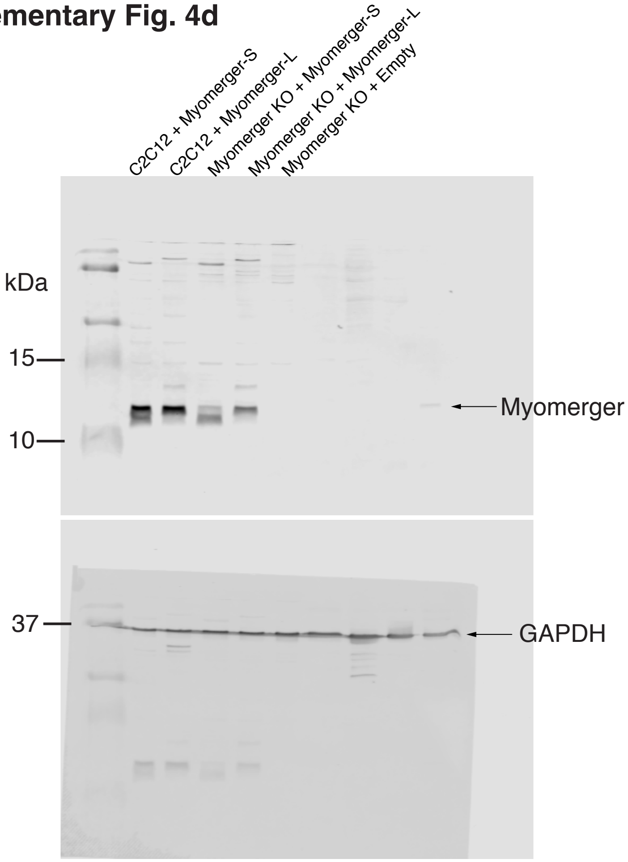

Supplementary Fig. 5a

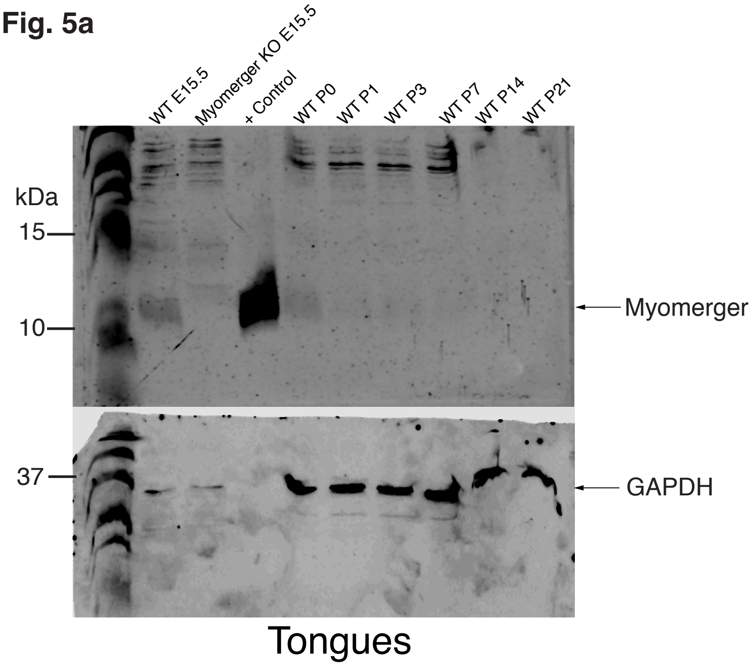

Supplementary Figure 6. Uncropped western blots from Figures 2 and 3, and Supplementary Figures 3, 4, and 5.

Supplementary Table 1. Expression of MyoD-regulated genes not expressed in fibroblasts

10T 1/2 Fibroblast RNAseq			Genes induced by MyoD in MEFs	
RPKM	Gene Symbol	Gene Symbol	logFC	Gene Title
0	Tnnt2	Tnnt2	9.17825573	troponin T2, cardiac
0	Tnnc2	Tnnc2	8.97110204	troponin C2, fast
0	Mylpf	Mylpf	8.85491471	myosin light chain, phosphorylatable, fast skeletal muscle
0	Myh3	Myh3	8.82920559	myosin, heavy polypeptide 3, skeletal muscle, embryonic
0	Actc1	Actc1	8.81751549	actin, alpha, cardiac muscle 1
0	Myog	Myog	8.80245903	myogenin
0.10269196	Tnni1	Tnni1	8.68606205	troponin I, skeletal, slow 1
0.17443733	Tmem8c	Tmem8c	8.41202322	transmembrane protein 8C
0	Tnnt3	Tnnt3	8.34000842	troponin T3, skeletal, fast
0	Acta1	Acta1	8.18874588	actin, alpha 1, skeletal muscle
0	Synpo2l	Synpo2l	7.92221429	synaptopodin 2-like
0	Tmem182	Tmem182	7.8533301	transmembrane protein 182
0	Myl4	Myl4	7.81495201	myosin, light polypeptide 4
0	Gm7325	Gm7325	7.74051592	predicted gene 7325
0	Mybph	Mybph	7.68579778	myosin binding protein H
0	Unc45b	Unc45b	7.5189577	unc-45 homolog B (C. elegans)
0	Chrng	Chrng	7.36205148	cholinergic receptor, nicotinic, gamma polypeptide
1.1548198	Tnnc1	Tnnc1	7.23204875	troponin C, cardiac/slow skeletal
0	Srl	Srl	7.12836254	sarcalumenin
0	Tnnt1	Tnnt1	7.07098707	troponin T1, skeletal, slow
0	Myl1	Myl1	6.91330584	myosin, light polypeptide 1
0	Lmod3	Lmod3	6.6883609	leiomodin 3 (fetal)
0	Smyd1	Smyd1	6.61346735	SET and MYND domain containing 1
0	Myom2	Myom2	6.60135439	myomesin 2
0	Tnni2	Tnni2	6.55667041	troponin I, skeletal, fast 2
0	Bex1	Bex1	6.50980382	brain expressed gene 1
0	Ttn	Ttn	6.46048441	titin
0.03008717	Ldb3	Ldb3	6.41795605	LIM domain binding 3
0	Casq2	Casq2	6.19397152	calsequestrin 2
0	Rbm24	Rbm24	6.11294525	RNA binding motif protein 24
0	Erbb3	Erbb3	6.10560693	v-erb-b2 erythroblastic leukemia viral oncogene homolog 3 (avian)
0	Ryr1	Ryr1	6.0652338	ryanodine receptor 1, skeletal muscle
0	Sgcg	Sgcg	5.92460516	sarcoglycan, gamma (dystrophin-associated glycoprotein)
0	Myh1	Myh1	5.85086114	myosin, heavy polypeptide 1, skeletal muscle, adult
0	Rtn2	Rtn2	5.78671503	reticulon 2 (Z-band associated protein)
0	Cdh15	Cdh15	5.75445913	cadherin 15
0	Srpk3	Srpk3	5.74459651	serine/arginine-rich protein specific kinase 3
0	Atp2a1	Atp2a1	5.73059525	ATPase, Ca++ transporting, cardiac muscle, fast twitch 1
0	Cox8b	Cox8b	5.70246683	cytochrome c oxidase subunit VIIIb
0	Neb	Neb	5.61038516	nebulin
0	Ckm	Ckm	5.56404176	creatine kinase, muscle
0.49870777	Stac3	Stac3	5.55938339	SH3 and cysteine rich domain 3
0	Myod1	Myod1	5.53329027	myogenic differentiation 1
0.14007927	Baiap2l1	Baiap2l1	5.52851946	BAI1-associated protein 2-like 1
0	Klhl30	Klhl30	5.49606225	kelch-like 30
0	Hfe2	Hfe2	5.47955255	hemochromatosis type 2 (juvenile) (human homolog)
0	Itgb1bp2	Itgb1bp2	5.46961023	integrin beta 1 binding protein 2
0	Myoz2	Myoz2	5.45057275	myozenin 2
0	Ccdc141	Ccdc141	5.43110797	coiled-coil domain containing 141
0.8347588	Rragd	Rragd	5.39364809	Ras-related GTP binding D
0.34770787	Dusp27	Dusp27	5.38264126	dual specificity phosphatase 27 (putative)
0	Ddc	Ddc	5.21600019	dopa decarboxylase
0	Cacna1s	Cacna1s	5.13392476	calcium channel, voltage-dependent, L type, alpha 1S subunit
0	Cap2	Cap2	5.11895139	CAP, adenylate cyclase-associated protein, 2 (yeast)
0	Arx	Arx	5.03676722	aristaless related homeobox
0	Trim54	Trim54	4.96547168	tripartite motif-containing 54
0	Myh7	Myh7	4.94561778	myosin, heavy polypeptide 7, cardiac muscle, beta
0	Smpx	Smpx	4.90407245	small muscle protein, X-linked
0	Fgf21	Fgf21	4.8725558	fibroblast growth factor 21
0	Ctrb1	Ctrb1	4.85885056	chymotrypsinogen B1
0	Igf2	Igf2	4.85000065	insulin-like growth factor 2
0	Des	Des	4.8392517	desmin
0	Mb	Mb	4.77738675	myoglobin
0	Sgca	Sgca	4.73764765	sarcoglycan, alpha (dystrophin-associated glycoprotein)
1.318527	Murc	Murc	4.72338191	muscle-related coiled-coil protein
0	Chrna1	Chrna1	4.69200919	cholinergic receptor, nicotinic, alpha polypeptide 1 (muscle)
0	Mylk4	Mylk4	4.68648371	myosin light chain kinase family, member 4
0	Rapsn	Rapsn	4.64866559	receptor-associated protein of the synapse
0	Myom1	Myom1	4.63484611	myomesin 1
0	Hspb2	Hspb2	4.62747615	heat shock protein 2
0	Pgam2	Pgam2	4.58691261	phosphoglycerate mutase 2
0.265493	Sytl2	Sytl2	4.55217229	synaptotagmin-like 2
0	Fxyd6	Fxyd6	4.52299238	FXYD domain-containing ion transport regulator 6
0.48914617	Rap1gap2	Rap1gap2	4.51901864	RAP1 GTPase activating protein 2
0.67196727	Itga7	Itga7	4.51236805	integrin alpha 7
0	Pvalb	Pvalb	4.47319646	parvalbumin
0	Prkag3	Prkag3	4.46661125	protein kinase, AMP-activated, gamma 3 non-catatlytic subunit
0	Ablim3	Ablim3	4.45809028	actin binding LIM protein family, member 3
0	Cdkn1c	Cdkn1c	4.42472929	cyclin-dependent kinase inhibitor 1C (P57)
0	Adssl1	Adssl1	4.40935794	adenylosuccinate synthetase like 1
0	Aldh1a7	Aldh1a7	4.4004752	aldehyde dehydrogenase family 1, subfamily A7
0.6663085	Dbndd1	Dbndd1	4.39086189	dysbindin (dystrobrevin binding protein 1) domain containing 1
0	Fndc5	Fndc5	4.38695116	fibronectin type III domain containing 5
0	Tm6sf1	Tm6sf1	4.32018565	transmembrane 6 superfamily member 1
1.2547097	H19	H19	4.30164594	H19, imprinted maternally expressed transcript
0	Dll1	Dll1	4.22362994	delta-like 1 (Drosophila)
0	Dhrs7c	Dhrs7c	4.19664777	dehydrogenase/reductase (SDR family) member 7C
1.399957	Cnr1	Cnr1	4.18134068	cannabinoid receptor 1 (brain)
0	Tspan33	Tspan33	4.17575099	tetraspanin 33
0	Hrc	Hrc	4.14582683	histidine rich calcium binding protein
0	Frmd4b	Frmd4b	4.14531439	FERM domain containing 4B
0	Inpp4b	Inpp4b	4.11722919	inositol polyphosphate-4-phosphatase, type II
0	Tceal7	Tceal7	4.04110915	transcription elongation factor A (SII)-like 7
0	Pde2a	Pde2a	4.02843986	phosphodiesterase 2A, cGMP-stimulated
1.1807443	Mfap3l	Mfap3l	4.01503916	microfibrillar-associated protein 3-like
0	Myom3	Myom3	4.00756366	myomesin family, member 3
0	Ppfia4	Ppfia4	3.99064545	protein tyrosine phosphatase, receptor type, f polypeptide (PTPRF), interacting protein (liprin), alpha 4
0	Tbc1d8	Tbc1d8	3.98645009	TBC1 domain family, member 8
0	C1qtnf3	C1qtnf3	3.92532533	C1q and tumor necrosis factor related protein 3
0	Cox6a2	Cox6a2	3.88010541	cytochrome c oxidase subunit VIa polypeptide 2

Supplementary Table 2. Primers used in this study

Pair	Description	For. Primer	Rev. Primer
1	Genotyping for *Gm7325* mutation	GAAGGGAGGACTCCACACCC	CGCCTGGACTAACCGGCTCC
2	Cloning Gm7325 locus containing short and long isoforms	AGTGATGCTGAATCCACCGCA	CCAATAACAACACACTGTCCT
3	Cloning of *Gm7325* - long	ATGCCAGAAGAAAGCTGCACTG	TCACTTCTGGGGGCCCAATCTC
4	Cloning of *Gm7325* - short	ATGCCCGTTCCATTGCTCCCGA	TCACTTCTGGGGGCCCAATCTC
5	myomerger-short SYBR	CAGGAGGGCAAGAAGTTCAG	ATGTCTTGGGAGCTCAGTCG
6	myomerger-long SYBR	ACCAGCTTTCATGCCAGAAG	ATGTCTTGGGAGCTCAGTCG
7	myomaker SYBR	ATCGCTACCAAGAGGCGTT	CACAGCACAGACAAACCAGG
8	Tm6sf1 SYBR	TTAGTGGTCCCTGGATGCTC	GACGCACCAATGTGAGAAAA
9	Tspan33 SYBR	GGGGACGAGTTCTCCTTCG	TGCTTCTGCGTGCTTCATTAG
10	Tmem182 SYBR	GGCTCTCTTCGGAGCTTTGG	GGTGGCTGATTGGTGTACCAG
11	Myogenin SYBR	CTACAGGCCTTGCTCAGCTC	GTGGGAGTTGCATTCACTGG
12	Ckm SYBR	ACCTCCACAGCACAGACAGA	CAGCTTGAACTTGTTGTGGG
13	Myh4 SYBR	GCAGGACTTGGTGGACAAAC	ACTTGGCCAGGTTGACATTG
14	GAPDH SYBR	TGCGACTTCAACAGCAACTC	GCCTCTCTTGCTCAGTGTCC
